# Characterizing Intimate Partner Violence-Caused Brain Injury in a Sample of Survivors in the Two Spirit, Lesbian, Gay, Bisexual, Transgender, Queer or Questioning Community

**DOI:** 10.1177/08862605241256390

**Published:** 2024-06-06

**Authors:** Tori N. Stranges, Rory A. Marshall, Rebecca Godard, Deana Simonetto, Paul van Donkelaar

**Affiliations:** 1 University of British Columbia—Okanagan, Kelowna, BC, Canada; 2 Alberta Health Services—Emergency Medical Services, Calgary, AB, Canada

**Keywords:** brain injury, intimate partner violence, sexual minority, 2S/LGBTQ health

## Abstract

Research in the field of intimate partner violence-caused brain injury (IPV-BI) has predominantly focused on heterosexual women, ignoring the unique needs of the Two Spirit, Lesbian, Gay, Bisexual, Transgender, Queer or Questioning (2S/LGBTQ) community. The purpose of this exploratory research was to better understand the prevalence of IPV and IPV-BI in 2S/LGBTQ relationships where IPV was defined as physical, psychological, financial, sexual, and/or identity-based abuse from a current of former intimate partner. This study used a cross sectional internet-based survey that ran from September to December of 2022. In addition to descriptive statistics, prevalence rates and their corresponding Wilson Score confidence intervals are reported to estimate the proportion of individuals who experienced IPV and IPV-BI. Finally, for both gender identity and sexual orientation, we tested whether participants with each identity had differing levels of brain injury severity compared to participants who did not hold that identity using Mann–Whitney *U* tests. In total, 170 2S/LGBTQ+ adults responded to the survey. Among the respondents, 54% identified as Two-Spirit, 24% identified as gay, 17% identified as queer, 14% identified as bisexual, and 8% identified as lesbian or pansexual, respectively. Respondents were predominantly multiracial, post-secondary educated, full-time employed, cisgender women (35%) or cisgender men (19%). The overwhelming majority reported lifetime prevalence of IPV at 98% (*n* = 166, 95% CI [94.11, 99.08]). Additionally, 68% (*n* = 115, 95% CI [60.29, 74.22]) of participants reported symptoms consistent with an IPV-BI. These results are consistent with the findings that the 2S/LGBTQ community are at heightened risk of experiencing physical IPV. These findings are the first to our knowledge to report a high rate of symptoms consistent with an IPV-BI in the 2S/LGBTQ population.

## Introduction

Intimate partner violence (IPV) is a public and personal health epidemic in Canada as 44% of people who identify as women have experienced IPV in their lifetime (Cotter, 2021). IPV is defined as a pattern of physical, sexual, psychological, and emotional violence in the context of coercive control by a former or current intimate partner (Humphreys & Campbell, 2010). Injuries from physical IPV vary from minor bruises and abrasions to multisystem trauma or death (Sun et al., 2023). Injuries to the face, head, and neck are the most common in physical IPV, primarily deriving from direct blunt trauma (e.g., punches, slaps, kicks), indirect blunt trauma (e.g., throwing into walls, pushing down stairs), and non-fatal strangulation (NFS; e.g., being “choked out”) (Sheridan & Nash, 2007). In the last two decades, research and public health efforts have highlighted that those who experience IPV, may also experience a brain injury (BI) as a result of the physical violence (J. K. Campbell et al., 2022; Esopenko et al., 2021; Haag et al., 2022; Hunnicutt et al., 2019; Valera & Berenbaum, 2003). A BI is defined as a complex pathophysiological process affecting the brain caused by external forces imparted on the body (Giza & Hovda, 2014). Silverberg et al. (2023) recently included an updated version of this definition to include an object striking the head, striking the head off an object, an acceleration/deceleration injury that does not include any object hitting the head (e.g., violently shaking), and explosive/blast injury leading to a BI. Interestingly, unlike BIs experienced by the general population, when characterizing those experienced by survivors/victims of IPV it is important to consider a hypoxic/anoxic injury via NFS. NFS-caused BI, although not historically thought of as a BI etiology, does result from external forces posing pathophysiological detriment to the brain (J. C. Campbell et al., 2018). Thus, IPV-caused BI (IPV-BI) can be uni- or multi-modal in nature, differing from other etiologies commonly seen in the BI literature (e.g., car accidents, sport injuries, workplace accidents) and is emerging as a widespread consequence of IPV.

Globally, it is estimated that 27% of women have experienced physical IPV in their lifetime (Sardinha et al., 2022). Estimates from the National Intimate Partner and Sexual Violence Survey indicate that 1 in 10 (11.6 million) U.S. women have been strangled by an intimate partner (Smith et al., 2017). Further, it has been reported that up to 92% of women have symptoms consistent with a BI following physical IPV (Zieman et al., 2017). Different from a bruise or broken bone, BI poses many unique challenges to an individual. Survivors who sustain a BI often report a constellation of physical, cognitive, emotional, and behavioral symptoms which can become chronic. Moreover, repeated trauma to the head, as seen in IPV, can result in symptoms such as fatigue, memory loss, confusion, aggression, and impaired judgment as well as physical comorbidities including chronic pain and cardiovascular, urological, gynecological, and pregnancy-related conditions (Stubbs & Szoeke, 2022). IPV can also lead to psychopathological comorbidities such as post-traumatic stress disorder, depression, and anxiety that can hide and/or exacerbate BI symptoms (Dillon et al., 2013). The insidious nature of IPV-BI paired with inadequate or absent pathways to recovery can lead to challenges in the ability to secure employment, maintain safe housing, and parent children. For example, when exploring the potential impact of IPV-BI on parenting disputes, Boyle et al. (2022) demonstrated the legal repercussions and vulnerability women would experience in the justice system. This was especially apparent when it came to decision-making about their capacity to parent after sustaining an IPV-BI.

Two-Spirit, Lesbian, Gay, Bisexual, Transgender, Queer or Questioning (2S/LGBTQ) is an umbrella term that includes individuals who have both diverse sexual orientations as well as gender identities. Two-Spirit is a term used across North America meant to facilitate Indigenous Peoples’ connections with nation-specific expressions, roles of gender, and sexual diversity. The myth that IPV in 2S/LGBTQ relationships is rare has been largely debunked in research. The Canadian Survey of Safety in Public and Private Spaces indicated almost 70% of sexual minority women have experienced IPV since the age of 15 (Jaffray, 2021). The 2S/LGBTQ community and those with a history of 2S/LGBTQ relationships are at a higher risk of experiencing psychological, physical, and sexual IPV relative to those who identify as heterosexual (Black et al., 2011; McLaughlin et al., 2012; Messinger, 2011; Porter & Williams, 2011). Further, when a 2S/LGBTQ person seeks help following an experience of IPV, their experiences are more likely to be taken less seriously and less likely to lead to a recommendation that the survivor leave the abuser (Sorenson & Thomas, 2009; Younglove et al., 2002). The reality for many survivors of IPV in 2S/LGBTQ relationships is the people they first turn to for help do not validate their experiences as legitimate abuse and/or refuse to help. This leads to survivors doubting whether they are true victims, deserve help, should try to leave, and whether help will be there for them if they do leave (Messinger, 2020). 2S/LGBTQ survivors face major barriers to reaching safety and accessing services in a world that stigmatizes their identities and overlooks their relationships.

Research in the field of IPV-BI has predominantly focused on heterosexual women, rather than sex and gender minority (SGM) 2S/LGBTQ community members. Similarly, IPV-BI has been largely ignored in the SGM literature. Gender-based violence interventions and public discourse surrounding IPV-BI have remained shaped by heteronormativity and cisnormativity, delegitimizing violence experienced in queer relationships. Despite previous research highlighting people who identify as part of the 2S/LGBTQ community are at heightened risk of experiencing IPV, no attention has been given to the rate and ways in which BI occurs (Messinger, 2011). The generalizability of much published research on this issue is problematic as 2S/LGBTQ people are more likely to experience *minority stress* in the form of intolerance, discrimination, harassment, and the threat of violence due to their gender identity and/or sexual orientation (Balsam & Szymanski, 2005; Brooks, 1981). Minority stress adds to the complexity of experiencing IPV-BI in a 2S/LGBTQ relationship and is generally not taken into account in research on IPV-BI due to the fact that it has been limited to the cisgender, heterosexual community.

Research has demonstrated that head trauma and attempted strangulation sustained in the context of IPV can result in a BI. However, no research exists for the 2S/LGBTQ community, indicating a gap in the knowledge on the prevalence of IPV-BI. The current study is the first to explore this issue. The objectives are to: (a) report that rates of symptoms consistent with an IPV-BI in 2S/LGBTQ people and (b) determine how these rates vary across subgroups of the 2S/LGBTQ population defined by gender identity and sexual orientation.

## Methods

### Ethics

This study was reviewed and approved by the Behavioural Research Ethics Board at the University of British Columbia Okanagan (H21-03256).

### Sample and Recruitment

To address the objectives of this work, an observational cross-sectional approach was selected. The online survey hosted on Qualtrics ran from September 2022 to December 2022. Eligibility criteria included self-identified English-speaking 2S/LGBTQ people (18+) living in British Columbia, Canada. Exclusion criteria included those who were under the age of 18 and resided outside of British Columbia. Entry into a $25 draw was offered to those who completed the survey. Survey and incentive responses were collected and stored separately to protect confidentiality.

Recruitment was conducted through local, regional, and provincial 2S/LGBTQ advocacy organizations through social media accounts, email listservs, and in-person flyers. Recruitment was also conducted through institutional, and personal social media accounts (Facebook, Instagram, Twitter, and Discord) through a direct link or QR code. In total, 2,391 responses were collected. All recruitment materials mentioned that the study was focused on 2S/LGBTQ brain health but was left intentionally vague around experiences of IPV and potential IPV-BI for participant safety. Participants provided informed consent and a content disclaimer about the difficult nature of the questions pertaining to IPV and IPV-BI before completing the survey.

### Evaluation of IPV and IPV-BI

#### Lifetime IPV History

Direct lifetime history of IPV was measured with one-item that asked, “Have you experienced intimate partner violence in your lifetime?” Participants were given the response option of “Yes” or “No.” IPV was defined as: “psychological, physical, sexual or identity-based abuse from an intimate partner either past or present.” The inclusion of identity-based abuse was made to reflect the particularities of IPV in 2S/LGBTQ+ relationships.

#### Brain Injury

A six-item measure of symptoms consistent with a BI was guided by the Brain Injury Severity Assessment (BISA) tool (Valera & Berenbaum, 2003). The BISA is typically delivered in a semi-structured interview format; however, that was not possible under the confines of survey research, thus adapting this established tool was necessary for this study. Affirmative or negative responses were employed, coded as 0 (no) or 1 (yes), to yield overall BISA scores ranging from 0 to 6 (0 = no symptoms of probable BI history, 1 to 6 = history consistent with BI history with a greater score indicating a higher severity).

#### Classification of Abuse

Types of IPV experienced and indirect lifetime history of IPV were measured using 14-items adapted from the Revised Sexual and Gender Minorities-Conflict Tactic Scale 2 (SGM-CTS2) (Dyar et al., 2021) to measure participants’ experience with physical abuse (four items), psychological abuse (also referred to as coercive control) (five items), financial abuse (one item), sexual abuse (two items), or identity-based abuse (two items) from a former or current intimate partner. This SGM-CTS2 was developed from the revised Conflict Tactic Scale (Straus et al., 1996) to include measures of specific IPV tactics experienced by SGM groups. This measure assesses a wide range of IPV tactics that are culturally sensitive and has demonstrated psychometric validity in a sample of 2S/LGBTQ+ people. Participants indicated if they experienced any of these forms of abuse through the response options of “Yes” or “No.” Physical abuse questions were cross-referenced with the BISA questions.

#### Demographics

Demographic information gathered included geographic location, race, Indigeneity, socioeconomic status, employment status, highest level of education, marital status, religious affiliation, number of dependents, sexual orientation, and gender identity. Given the context of this research, respondents were able to select multiple responses for gender and/or sexual orientation as well as race/ethnicity.

### Data Analysis

#### Data Cleaning

Responses were compared against the inclusion criteria for initial validation screening. As guaranteed or possible compensation is an attraction for potentially fraudulent responses, a data cleaning protocol was used to ensure data integrity through response cleaning (Griffin et al., 2021). This protocol included: (a) confirming the respondent’s geographic location based on the first three digits of their postal code provided (27% of initial data set remained, *n* = 651), (b) removing respondents who completed less than 60% of the survey (21% of initial data set remained, *n* = 509), (3) removing qualitative duplicates (exact duplicate of qualitative responses, indicative of bots) and conflicting data (refers to inconsistencies, discrepancies, or contradictions within the dataset, for example a non-indigenous person selecting Two-Spirit) were removed (17% of initial data set remained, *n* = 403), and, finally, (4) IP address verification leaving 7% of the initial dataset (*N* = 170) for analysis. Among these 170 included participants, missing data were rare (2.5%).

#### Analysis

Descriptive statistics were obtained using SPSS Statistics (Version 29.0. Armonk, NY: IBM Corp.) and OpenEpi (Version 3.01). We calculated prevalence rates and their corresponding Wilson Score confidence intervals to estimate the proportion of individuals who experienced IPV and symptoms consistent with an IPV-BI. Further quantitative analyses were conducted using RStudio (Boston, MA). For both gender identity and sexual orientation, we tested whether participants with each gender identity and sexual orientation had differing levels of suspected BI severity compared to participants who did not hold that identity or orientation. Due to the small sample size and non-normal distribution of potential BI scores (skew = 0.11, kurtosis = −1.60), we used Mann–Whitney *U* tests, which are robust to violations of normality (Divine et al., 2013). Achieved power to detect medium effects ranged from 31% to 58% based on group size. Despite the relatively low achieved power, these tests remained important because of the lack of research on IPV-BI in the 2S/LGBTQ population, the known difficulty in sampling the 2S/LGBTQ population, and the importance of acknowledging the complexity of 2S/LGBTQ experiences that vary by gender identity and sexual orientation (Ghorbanian et al., 2022; Hughes et al., 2021).

## Results

Table 1 shows demographic characteristics of the respondents. Mean age of the sample was 30 years (*SD* = 8.4). Among the respondents, 62% (*n* = 104) were Indigenous, and 54% (*n* = 92) identified as Two-Spirit. Across all participants, 24% (*n* = 41) identified as gay, 17% (*n* = 28) identified as queer, 14% (*n* = 24) identified as bisexual, and 8% (*n* = 14) identified as lesbian or pansexual, respectively. Respondents were predominantly multiracial, post-secondary educated, full time employed, cisgender-women (35%; *n* = 59), or cisgender-men (19%; *n* = 33).

**Table 1.** Demographic Information.

Indigeneity	
None	32.9% (*n* = 56)
First nation	43.5% (*n* = 74)
Metis	7.0% (*n* = 12)
Inuit	4.1% (*n* = 7)
Indigenous outside Turtle Island	5.3% (*n* = 9)
Prefer to self-describe (cree, mixed, caucasian)	2.4% (*n* = 4)
Two-spirit identity	
Yes	54.1% (*n* = 92)
No	42.9% (*n* = 73)
Intersex	
Yes	46.5% (*n* = 79)
No	45.3% (*n* = 77)
Unsure	7.1% (*n* = 12)
Gender identity	
Cis-woman	34.7% (*n* = 59)
Trans-woman	7.7% (*n* = 13)
Cis-man	19.4% (*n* = 33)
Trans-man	11.2% (*n* = 19)
Non-binary	4.2% (*n* = 7)
Genderqueer/Gender Nonconforming	7.7% (*n* = 13)
Sexual orientation	
Gay	24.1% (*n* = 41)
Lesbian	8.2% (*n* = 14)
Bisexual	14.1% (*n* = 24)
Pansexual	8.2% (*n* = 14)
Asexual	1.2% (*n* = 2)
Queer	16.7% (*n* = 28)
Straight	1.2% (*n* = 2)
Prefer to self-describe (omnisexual)	0.6% (*n* = 1)
Race/Ethnicity	
Non-indigenous	32.9% (*n* = 56)
White	67.1% (*n* = 114)
Black	5.3% (*n* = 9)
East/South Asian	3.5% (*n* = 6)
Latino	2.4% (*n* = 4)
Indigenous	67.1% (*n* = 114)
White	56.5% (*n* = 98)
Black	5.9% (*n* = 10)
East/South Asian	1.2% (*n* = 2)
Latino	2.4% (*n* = 4)
Education	
Less than high school	1.8% (*n* = 3)
Highschool diploma	30.0% (*n* = 51)
Trades certificate/Diploma	14.1% (*n* = 24)
College or CEGEP (collège d’enseignement général et professionnel)	15.9% (*n* = 27)
University certificate	12.9% (*n* = 22)
University bachelor degree	15.9% (*n* = 27)
Above bachelor degree	7.7% (*n* = 13)
Net income (before tax)	
<$30,000	18.2% (*n* = 31)
$30,000 to <$40,000	30.6% (*n* = 52)
$40,000 to <$50,000	17.1% (*n* = 29)
$50,000 to <$60,000	8.2% (*n* = 14)
$60,000 to <$80,000	12.4% (*n* = 21)
$80,000 to <$100,000	7.7% (*n* = 13)
$100,000 to <$150,000	3.5% (*n* = 6)
$150,000 or more	1.8% (*n* = 3)
Employment status	
Full time	70.6% (*n* = 120)
Part time	15.9% (*n* = 27)
Contract/Temporary	5.3% (*n* = 9)
Unemployed	0.6% (*n* = 1)
Unable to work	3.5% (*n* = 6)
Prefer to self-describe (student, retired)	3.5% (*n* = 6)
Marital status	
Married	18.2% (*n* = 31)
Common law	14.7% (*n* = 25)
Divorce	11.2% (*n* = 19)
Separated	10.0% (*n* = 17)
Single	38.8% (*n* = 66)
Dependants	
No dependants	32.4% (*n* = 55)
1	22.4% (*n* = 38)
2 or 3	31.2% (*n* = 53)
More than 4	13.5% (*n* = 23)

*Note.* Respondents were given the opportunity to select none or multiple for each of the demographic questions.

When asked directly, 84% (*n* = 143, 95% CI [77.88, 88.85] reported a history of IPV. By contrast, when responding to the SGM-CTS 2 questions, 98% (*n* = 166, 95% CI [94.11, 99.08]) indirectly screened positive for a history of IPV. Finally, results from the questions about IPV-BI showed that 68% (*n* = 115, 95% CI [60.29, 74.22]) screened positive for experiencing one or more episodes of IPV that resulted in symptoms consistent with a BI (BISA score >0).

Table 2 shows the cross tabulation of BISA scores across gender identity. This table indicates trans women were the most likely to experience IPV episodes resulting in a potential BI (81.8%; *n* = 36) and trans men were the least likely (60.6%; *n* = 40). Table 3 shows the cross tabulation of the BISA scores across sexual orientation. This table indicates that queer people were the most likely to experience IPV episodes resulting in a potential BI (88.5%; *n* = 46) and bisexual people were the least likely (56.4%; *n* = 22). Table 4 reports the mean, median, standard deviation, skew, and kurtosis for all the BISA scores across the gender identity and sexual orientation subgroups.

**Table 2.** Brain Injury Severity Assessment Scores Cross Tabulated for Gender Identity.

BISA	Trans women	Cis women	Cis men	Non-binary	Queer	Trans men
BISA score						
Negative (0)	8	3	5	7	12	26
Positive (>0)	36	11	14	19	23	40
Total	44	14	19	26	35	66
% Greater than 0	81.8	78.6	73.7	73.1	65.7	60.6

*Note*. A score from 1 to 6 indicates an increasing severity of brain injury symptomology. Participants could select multiple gender identities. BISA = Brain Injury Severity Assessment

**Table 3.** Brain Injury Severity Assessment Scores Cross Tabulated for Sexual Orientation.

BISA	Queer	Asexual	Lesbian	Gay	Pansexual	Straight	Bisexual
BISA score							
Negative (0)	6	1	9	17	9	1	17
Positive (>0)	46	4	24	44	18	2	22
Total	52	5	33	61	27	3	39
% Greater than 0	88.5	80.0	72.7	72.1	66.7	66.7	56.4

*Note*. A score from 1 to 6 indicates an increasing severity of brain injury symptomology. Participants could select multiple sexual orientations. BISA = Brain Injury Severity Assessment.

**Table 4.** Descriptive Statistics for BISA Scores across Gender Identity and Sexual Orientation Subgroups.

Variables	*n*	Mean	Median	*SD*	Skew	Kurtosis
Gender						
Cisgender men	19	2.32	2	2.14	0.42	1.82
Cisgender women	14	3.29	4	2.64	−0.12	1.22
Non-binary	26	3.69	4.5	2.54	−0.51	1.62
Gender-queer	35	2.83	3	2.57	0.10	1.30
Trans men	66	2.39	1	2.54	0.43	1.43
Trans women	44	3.30	3	2.13	−0.18	1.91
Sexual orientation						
Gay	61	2.67	3	2.17	0.17	1.77
Lesbian	33	2.97	2	2.65	0.11	1.20
Bisexual	39	2.44	2	2.48	0.26	1.39
Pansexual	27	2.74	4	2.49	0.05	1.27
Asexual	5	3.20	4	2.59	−0.17	1.40
Queer	52	3.52	4	2.40	−0.17	1.33
Straight	3	3.33	4	3.06	−0.21	1.50

*Note.* BISA = Brain Injury Severity Assessment.

**Figure 1.**
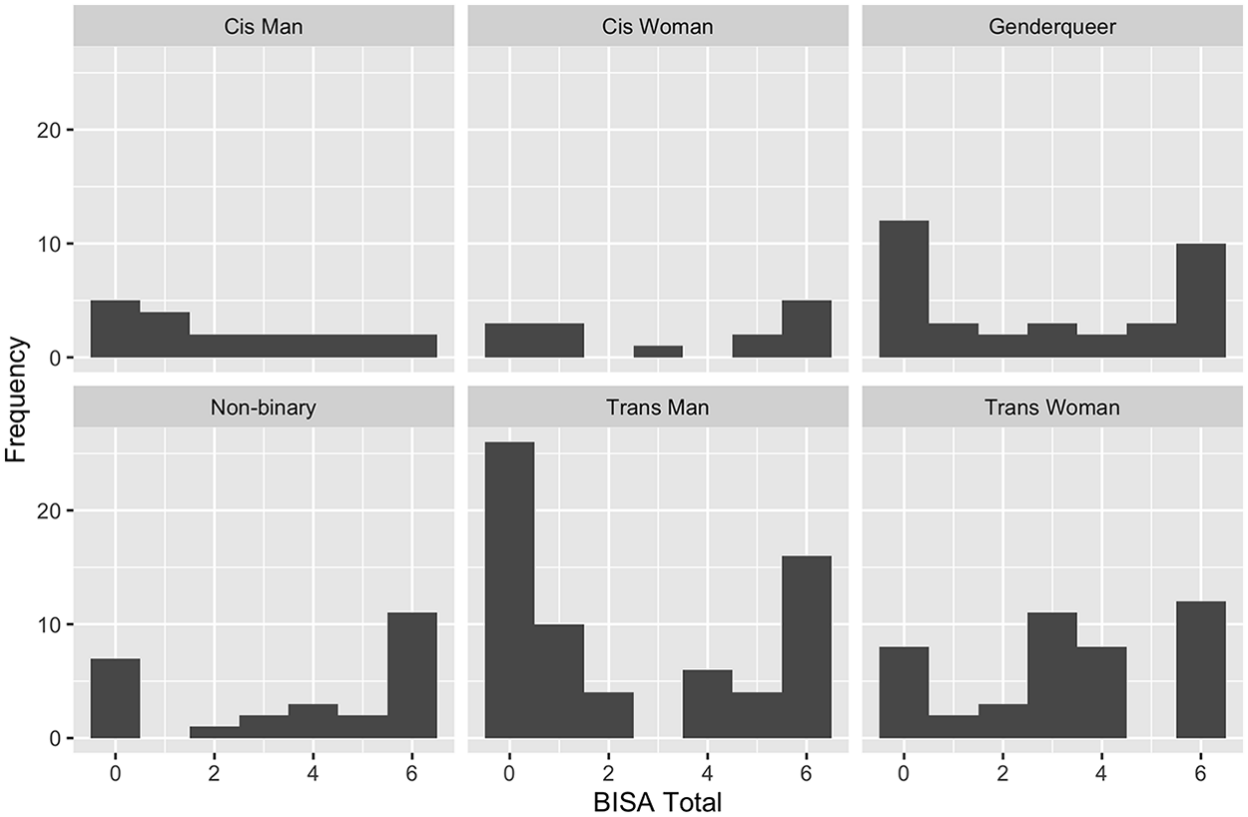
BISA score distribution across gender identity categories. BISA = Brain Injury Severity Assessment.

For gender identity, trans men (*n* = 66) had significantly lower exposure to potential BI compared to participants of other gender identities (*n* = 104, *W* = 2,787, *p* = .04, *d* = −0.31, location difference = −1.00, 95% CI [−2.00, −0.00]). No other gender identities had significantly different levels of exposure to potential BI (see Figure 1).

**Figure 2.**
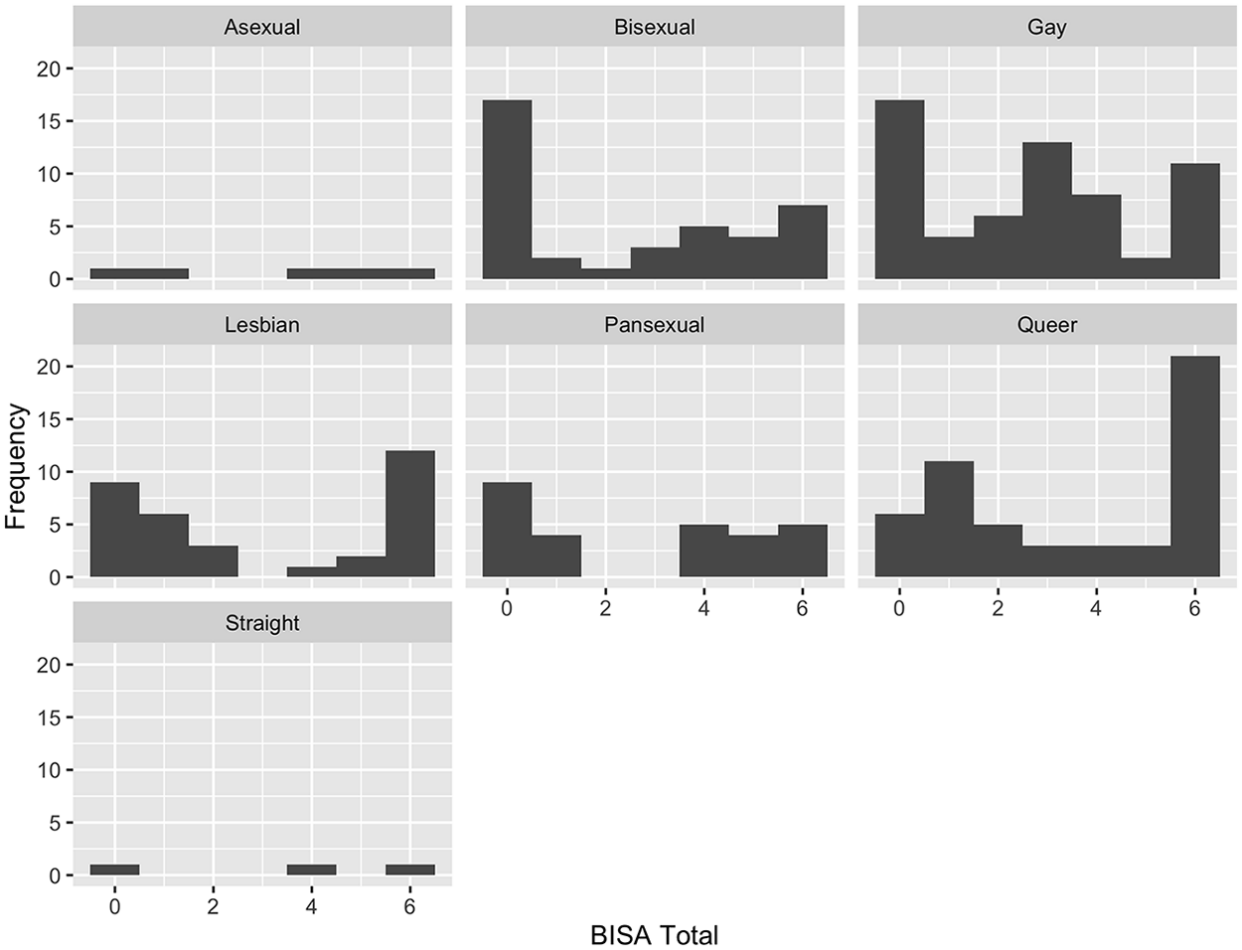
BISA score distribution across sexual orientation categories. BISA = Brain Injury Severity Assessment.

For sexual orientation, comparison of the levels of exposure to potential BI for asexual (*n* = 5) or straight (*n* = 3) participants were not completed due to small sample sizes. Participants who identified as queer (*n* = 52) had significantly higher levels of exposure to potential BI compared to participants of other sexual orientations (*n* = 118, *W* = 3,841, *p* = .008, *d* = 0.39, location difference = 1.00, 95% CI [0.00, 2.00]). No other sexual orientations had significantly different levels of exposure to potential BI (see Figure 2).

## Discussion

This study identified the occurrence of self-reported IPV and symptoms consistent with an IPV-BI among a sample of 2S/LGBTQ people residing in British Columbia, Canada. Almost our entire sample (98%) reported lifetime prevalence of IPV and ~70% of participants reported symptoms consistent with an IPV-BI. Hunnicutt et al. (2019) arrived at similar findings when they sought to explore IPV-BI in a sample of predominantly heterosexual (88.5%) online survey respondents using the HELPS Brain Injury Screening Tool. This study revealed that 92% of the survey respondents had experienced some type of IPV in their most recent violent relationship and 49% of the survey respondents were “at risk for TBI” based on the criteria from the HELPS questionnaire. While both this study and the current study used different screening tools (HELPS vs. BISA), the results are consistent with the literature that suggest a large proportion of survivors, regardless of their gender identity and sexual orientation, may be subject to IPV and subsequent IPV-BI.

Moreover, our findings shed light on the depth of these experiences. When participants were asked explicit questions about the types of violence experienced in their relationships using the SGM-CTS 2, 98% of respondents expressed experiencing some type of IPV in their lifetime compared to 84% when asked to self-report more generally: “*Have you experienced intimate partner violence in your lifetime?*” This indicates that almost 15% of survivors did not recognize they had experienced IPV when asked to self-report their experiences. These results are consistent with the findings that survivors of IPV are unlikely, unwilling, or unable to report violence unless directly asked (Haag et al., 2022). This may be further compounded in 2S/LGBTQ relationships where societal stigma, fear of discrimination, and concerns about disclosure within a potentially unsupportive environment can act as significant barriers to reporting. Similarly, participants from a qualitative study of experiences of lesbian, bisexual, and trans survivors of IPV reported that the lack of information about IPV in queer relationships left them unprepared to handle abuse when it happened (Bornstein et al., 2006). This may be due to homophobia, biphobia, transphobia, and a lack of positive, public role models for healthy 2S/LGBTQ relationships that further contributes to a heightened risk of experiencing IPV.

Throughout the literature, the recurring model of IPV—in which cisgender, heterosexual men are perpetrators, cisgender heterosexual women are victims, and patriarchy and/or heterosexism is considered the main underlying cause—contributes to the difficulty of defining a queer partner’s behavior as IPV (Calton et al., 2016). IPV in 2S/LGBTQ relationships poses a distinct challenge, primarily due to the potential invisibility of the relationship itself. For example, in a same-sex relationship where the couple is not out, IPV may go unnoticed by friends, family, or professionals, making it difficult for the survivor to seek help or leave. This raises a paradox of legitimizing one’s experience of abuse within a relationship ostensibly perceived as nonexistent. This is exacerbated by the historical predominance of IPV discourse within the framework of cisgender, heterosexual relationships, which has led to the marginalization of IPV in 2S/LGBTQ relationships. Consequently, individuals in 2S/LGBTQ relationships may, by extension, struggle to fully acknowledge or comprehend the nature of the IPV they are experiencing.

IPV is often cyclic and escalating (Krug et al., 2002). It is well documented that experiences of IPV may lead to complex, overlapping psychiatric, cognitive, psychological, and physical conditions that may have similar symptom presentations to IPV-BI. However, using a survey guided by the BISA, this study demonstrated that 2S/LGBTQ survivors of IPV may often experience symptoms indicative of a BI and thus may be susceptible to these complex challenges. These results are consistent with the findings that the 2S/LGBTQ community are at heightened risk of experiencing physical IPV and that IPV-BI may happen in the 2S/LGBTQ community at alarming rates (Black et al., 2011; Jaffray, 2021; McLaughlin et al., 2012; Messinger, 2011; Porter & Williams, 2011).

IPV has been historically misunderstood as less prevalent and less severe in 2S/LGBTQ relationships. Messinger (2011) posits that binary social constructions of gender shape societal understandings of IPV in 2S/LGBTQ relationships. By way of illustration, within a heterosexual framing, cisgender men embody dominant hegemonic masculine traits such as strength, dominance, and aggression. Society perceives that to be “normal” and thus violence is expected. In 2S/LGBTQ relationships, individuals perform femininities and masculinities that disrupt our normative understanding of IPV. For example, a gay man may perform a mix of masculine and feminine roles and be seen as more feminine than a straight man who performs traditional masculine roles and identities. As such, one may be less likely to expect a gay man to be physically violent because he is not adhering to traditional ways men perform masculinity by appearing too feminine. To further this point, gender is not a fixed characteristic but rather something that is continually constructed and performed through our daily actions (Butler, 1990; West & Zimmerman, 1987). The way gender expectations are ascribed in society has consequences in how IPV is understood as well as who are perpetrators and victims. This challenges the popular notion that IPV is predominantly perpetrated by cisgender, heterosexual men and reinforces that IPV and IPV-BI also occurs in 2S/LGBTQ relationships. Within this framing, those with diverse identities can perpetrate and be victims of IPV and IPV-BI.

In this study, there was overwhelming representation among each subgroup of gender identity and sexual orientation. As such, a subgroup analysis was completed to compare the prevalence of experiencing symptoms consistent with an IPV-BI by gender identity and sexual orientation. For gender identity, trans men were the least likely to experience IPV episodes resulting in a potential BI compared to all other identities and trans women were the most likely. For sexual orientation, queer were the most likely to experience IPV episodes resulting in a potential BI compared to all other orientations and reported the greatest number of symptoms. This is no surprise as previous research has demonstrated that the 2S/LGBTQ community is at an increased risk of experiencing physical IPV—particularly the gender diverse (queer) community and trans women of color (Sherman et al, 2022). Interestingly, previous research has demonstrated that bisexual people, specifically bisexual women, have the most risk of experiencing violence compared to other members of the 2S/LGBTQ community (Coston, 2021; Johnson & Grove, 2017). These preliminary findings demonstrate that bisexual people are the least likely subgroup to report symptoms consistent with an IPV-BI. Further research is needed to better understand IPV-BI variation across gender identity and sexual orientation.

Among the respondents, ~60% were Indigenous, and ~55% identified as Two-Spirit. Of the Indigenous respondents, 67% (*n* = 98) also identified as white which may have been due to not including Indigenous as an option in the race/ethnicity question or it may indicate participants were biracial. This is an overrepresentation as 6% of the population in the region is Indigenous (Statistics Canada, 2017). This is consistent with the literature that repeatedly concludes much of the elevated risk for experiencing IPV and IPV-BI is linked to colonization and related contextual factors such as reduced access to social resources (Brownridge, 2008; Daoud et al., 2013; Pedersen et al., 2013). Further, the overrepresentation of Two-Spirit respondents may be a result of intersecting oppressions (e.g., racism and/or colonialism intersecting with homophobia and/or transphobia). This highlights the need for a decolonized, strength-based, and collaborative approach to studying IPV and IPV-BI with Indigenous communities to better understand the systemic barriers they face (Maldonado-Rodriguez et al., 2021).

The struggle to legitimize 2S/LGBTQ IPV as a genuine public health concern may be rooted in the struggle to legitimize 2S/LGBTQ human rights. After all, if 2S/LGBTQ people are not afforded equal rights in many parts of North America and across the world, it should not be surprising that policies, services, funding, and research do not adequately reflect the concerns of 2S/LGBTQ people experiencing IPV (Messinger, 2020). This connection may underscore how discrimination, social stigma, and a lack of legal recognition can create hostile environments for 2S/LGBTQ community members both in private and public spaces. Lack of recognition and protection may lead to disparities in how IPV and IPV-BI within the 2S/LGBTQ community are addressed and understood. It may discourage individuals from seeking help or reporting abuse, as they may fear discrimination or judgment from authorities or service providers who may not understand their unique experiences. It may also result in a lack of dedicated resources and funding for research and support services tailored to the specific needs of the community. To address these issues, it is essential not only to work toward the inclusion of 2S/LGBTQ in understanding IPV and IPV-BI but also full recognition of 2S/LGBTQ human rights.

This research does not come without limitations. At this time, no survey decision-making tool is available to diagnose an IPV-BI (Dams-O’Connor et al., 2023). As such, this study relied on retrospective self-reports of BI signs and symptoms developed from a semi-structured interview tool (BISA). The findings reported here represent symptoms consistent with an IPV-BI as suspected IPV-BI was not adequately assessed using this tool. Further, it is important to acknowledge that people who have IPV histories and histories of stigma and discrimination based on their sexual orientation, gender identity, and gender expression experience complex and overlapping psychiatric, neurological, and functional changes as a result of their experience (e.g., PTSD, anxiety, depression, substance use, trauma) (Galovski et al., 2021). This creates a limitation in assessing BI in IPV contexts given the overlapping symptoms IPV survivors experience. Future studies should consider using a validated screening method for IPV-BI such as the brain injury screening questionnaire IPV (BISQ-IPV) module (Dams-O’Connor et al., 2023). Further, the respondents were from a single province in Canada predominately recruited from 2S/LGBTQ advocacy organizations which may have a potential for a bias sample. Thus, extending the generalizability of these results to all of Canada’s 2S/LGBTQ community must be done with caution. Pan-Canadian examination of our observations should be pursued for validation. Finally, research on the 2S/LGBTQ community has been critiqued for painting the diverse expressions of sexual orientation and gender identity with a monolithic brush, forgoing the nuance between subgroups of the 2S/LGBTQ community. The small numbers of participants with specific 2S/LGBTQ gender identities and sexual orientations made comparing rates of IPV-BI between groups challenging, and our estimates have wide confidence intervals. Future research with larger samples is needed to estimate the prevalence and severity of IPV-BI more precisely among subgroups within the 2S/LGBTQ community, particularly those identified within our research as potentially being at higher risk (i.e., transgender women and those with a queer sexual orientation). Although we acknowledge this as a limitation, our approach still provided more nuance and accuracy compared to those that collapse across all identities within the highly diverse 2S/LGBTQ community.

## Conclusion

In this study of 2S/LGBTQ people, 98% reported experiencing IPV. The vast majority (68%) of physical IPV respondents may have also endured an IPV-BI. This is, to our knowledge, the first study of its kind to characterize exposure to potential BIs from IPV in the 2S/LGBTQ community. This study reveals the pervasiveness of IPV and symptoms consistent with an IPV-BI in the 2S/LGBTQ community underscoring a critical public health crisis. The nuanced understanding of gender and the performance of gender roles within 2S/LGBTQ relationships challenge traditional frameworks of IPV, necessitating a broader conceptualization that includes diverse gender identities and sexual orientations. The disparities in IPV-BI exposure across different subgroups within the 2S/LGBTQ community further emphasize the need for tailored approaches in research, policy, and practice. Particularly, the overrepresentation of Indigenous and Two-Spirit individuals in our sample points to the intersectional nature of oppression and the urgent need for decolonized and inclusive research methodologies and health interventions. Findings suggest that 2S/LGBTQ people who experience IPV should be screened for a potential BI. This will help clinicians to better target their examinations, treatment, and referrals. It is hoped that the current findings will inform screening, assessment, and treatment for 2S/LGBTQ people by increasing awareness and encouraging further research and inquiry into this important yet understudied topic. Addressing these issues requires a concerted effort to dismantle systemic barriers, expand our understanding of IPV beyond heteronormative paradigms, and ensure equitable access to resources and support for all survivors of IPV.

## Supplemental Material

sj-docx-1-jiv-10.1177_08862605241256390 – Supplemental material for Characterizing Intimate Partner Violence-Caused Brain Injury in a Sample of Survivors in the Two Spirit, Lesbian, Gay, Bisexual, Transgender, Queer or Questioning Community Supplemental material, sj-docx-1-jiv-10.1177_08862605241256390 for Characterizing Intimate Partner Violence-Caused Brain Injury in a Sample of Survivors in the Two Spirit, Lesbian, Gay, Bisexual, Transgender, Queer or Questioning Community by Tori N. Stranges, Rory A. Marshall, Rebecca Godard, Deana Simonetto and Paul van Donkelaar in Journal of Interpersonal Violence
