# Supplementary material for: Characterizing Intimate Partner Violence-Caused Brain Injury in a Sample of Survivors in the Two Spirit, Lesbian, Gay, Bisexual, Transgender, Queer or Questioning Community
Source: J Interpers Violence. 2024 Jun 6;40(3-4):906–27. doi: 10.1177/08862605241256390 (PMC11673306; doi:10.1177/08862605241256390)
Supplement: sj-docx-1-jiv-10.1177_08862605241256390 – Supplemental material for Characterizing Intimate Partner Violence-Caused Brain Injury in a Sample of Survivors in the Two Spirit, Lesbian, Gay, Bisexual, Transgender, Queer or Questioning Community [file sj-docx-1-jiv-10.1177_08862605241256390.docx]

**Supplementary Material A. Online Survey Questions**

**Age (years)**:

Respecting that Indigenous peoples have their own gender and sexual expressions, roles, and identities, we have a few questions that are only for Indigenous people.

Do you identify as?

- First Nation
- Metis
- Inuit
- Indigenous to areas outside of Turtle Island (for example: Aboriginal, Torres Straight etc.)
- Prefer to self- describe (please specify): _____
- Prefer not to say
- None

Do you identify as Two-Spirit?

- Yes
- No

**Highest Level of Education**

What is the highest certificate, diploma or degree that you have completed?

- Less than high school diploma or its equivalent
- High school diploma or a high school equivalency certificate
- Trades certificate or diploma
- College, CEGEP or other non-university certificate or diploma (other than trades certificates or diplomas)
- University certificate or diploma below the bachelor's level
- Bachelor's degree (e.g., B.A., B.A. (Hons), B.Sc., LL.B.)
- University certificate, diploma or degree above the bachelor's level

**Which of the following best describes you (choose all that apply)**:

- Black
- East/ South Asian
- Latino
- Middle Eastern
- South Asian
- White
- Prefer to self-describe: ______

**Total Net Personal Income (before tax in the last year)**:

- Less than $30,000
- $30,000 to less than $40,000
- $40,000 to less than $50,000
- $50,000 to less than $60,000
- $60,000 to less than $80,000
- $80,000 to less than $100,000
- $100,000 to less than $150,000
- $150,000 or more
- Prefer not to answer

What is your employment status?

- Full-time
- Part-time
- Contract/ Temporary
- Unemployed
- Unable to work
- Prefer to self-describe: ____
- Prefer not to answer

What is your marital status?

- Married
- Common Law
- Divorced
- Separated
- Widowed
- Single
- Prefer not to answer

How many dependents do you have?

- No dependents
- 1
- 2-3
- More than 4
- Prefer not to answer

What religious family do you belong to or identify yourself most close with?

- Christian (Catholic, Protestant or any other Christian denomination)
- Buddhist
- Hindu
- Muslim
- Jewish
- Sikh
- No religion
- Prefer not to answer
- Prefer to self describe: ____

**Assigned sex at birth**:

What sex were you assigned at birth?

Male

Female

Something else

Prefer not to answer

Were you born with a variation in your physical sex characteristics? This is sometimes called being intersex or as having a difference in sex development (“DSD”). An official diagnosis is not required and please answer regardless of when you/others became aware of this innate physical variation (e.g., birth or later life).

Yes

No

Unsure

Prefer not to answer

**Current gender identity**:

How do you describe your gender identity? (Please check all that apply.)

- Man
- Woman
- Non- Binary
- Genderqueer/Gender non-conforming (GNC)

Prefer not to answer

- Prefer to self-describe:

**Sexual orientation identity**:

How do you describe your sexual orientation? (Please check all that apply.)

- Gay
- Lesbian
- Bisexual
- Pansexual
- Asexual
- Queer
- Straight

Prefer not to answer

- Prefer to self-describe:

How would you describe your current or most recent relationship?

- Monogamous
- Polyamorous
- Polygamous

Prefer not to answer

- Prefer to self-describe:

**Geographic location:**

Where is your physical location? Please provide the first 3 digits of your postal code.

- Open Text Box

**Lifetime intimate partner violence history**

Using the previous definitions for reference, have you experienced intimate partner violence in your lifetime? (Psychological, physical, financial, sexual or identity-based abuse)

- Yes
- No

**LGBT Perpetration**

If you have experienced intimate partner violence, how did this partner or partners describe their sexual orientation? Select all that apply.

- Gay
- Lesbian
- Bisexual
- Pansexual
- Asexual
- Queer
- Straight

Not sure or don’t know

Prefer not to answer

- Not stated (please state):
- Not applicable

If you have experienced intimate partner violence, how did this partner or partners describe their gender identity? Select all that apply.

- Cis man
- Cis woman
- Trans man/ Trans male
- Trans woman/ Trans female
- Two- spirit
- Non- binary
- Genderqueer/Gender non-conforming (GNC)

Not sure or don’t know

Prefer not to answer

- Not stated (please state):
- Not applicable

**Type of violence (modified SGM- CTS2 Dyar et al., 2021)**

My partner called me names, insulted me, or treated me disrespectfully.

- Yes
- No

My partner belittled me or made me feel ashamed or inadequate.

- Yes
- No

My partner threatened to hit or throw something at me.

- Yes
- No

I had a sprain, bruise, or small cut because of a fight with a partner.

- Yes
- No

I felt physical pain the next day because of a fight with a partner.

- Yes
- No

My partner physically put their hands around my neck with intent to cause harm.

- Yes
- No

My partner insisted on having sex or used threats when I did not want to (but did not use physical force).

- Yes
- No

My partner has used force (like hitting, holding down, or using a weapon) to make me have sex.

- Yes
- No

My partner monitored my time and made me account for my whereabouts.

- Yes
- No

My partner controlled or limited my access to money and other financial resources.

- Yes
- No

My partner made it difficult for me to see my friends, biological or chosen family*.

- Yes
- No

**Individuals who are not biologically or legally related who deliberately choose to support and nurture each other.*

My partner threatened to hurt themself or commit suicide when they were upset with me.

- Yes
- No

My partner ''outed'' me or threatened to “out” me --to friends or family, at school, work, or in other social circles.

- Yes
- No

My partner threatened to turn people in the queer community against me, or spread rumors about me in the community.

- Yes
- No

**Brain injury incidence- modified from BISA (Straus et al., 1996)**

After anything physical your partner ever did to you, did you ever lose consciousness or black out?

- Yes
- No

After anything physical your partner ever did to you, did you ever have a headache or head pain?

- Yes
- No

After anything physical your partner ever did to you, did you feel dizzy?

- Yes
- No

After anything physical that your partner ever did to you, did you ever feel stunned, really disoriented or have the sense of not knowing where you were or what time it was?

- Yes
- No

After anything physical that your partner ever did to you, did you ever "see stars or spots"?

- Yes
- No

After anything physical that your partner ever did to you, did you ever have any problems remembering things that happened right before or after that happened?

- Yes
- No

Is there anything else we missed, or you would like to share about your experience? (Open text box)
